# Morphological Study of Isolated Ovarian Preantral Follicles Using Fibrin Gel Plus Platelet Lysate after Subcutaneous Transplantation

**DOI:** 10.22074/cellj.2015.521

**Published:** 2015-04-08

**Authors:** Ali Reza Rajabzadeh, Hossein Eimani, Homa Mohseni Koochesfahani, Abdol-Hossein Shahvardi, Rouhollah Fathi

**Affiliations:** 1 Department of Embryology at Reproductive Biomedicine Research Center, Royan Institute for Reproductive Biomedicine, ACECR, Tehran, Iran; 2 Department of Cell and Molecular Biology, Faculty of Biological Sciences, Kharazmi University, Tehran, Iran; 3 Department of Anatomy, Faculty of Medicine, Baqiyatallah (a.s.) University of Medical Sciences, Tehran, Iran

**Keywords:** Transplantation, Follicle, Ovary

## Abstract

**Objective:**

Ovarian and follicle transplantation may preserve fertility in young cancer survivors. In this study, we have transplanted preantral follicles using fibrin gel as a carrier and fibrin gel supplemented with platelet lysate (PL) as a rich source of angiogenic and growth factors. The purpose of this study was to evaluate the role of fibrin gel and PL in follicle transplantation.

**Materials and Methods:**

In this experimental study, ovaries were taken from 14-day- old Naval Medical Research Institute (NMRI) mice. Preantral follicles were dissected from the ovaries and encapsulated into fibrin gel supplemented with 5, 10, 15 or 20% PL, then transplanted back into the same donor mice. Fibrin gels supplemented with PL that contained preantral follicles were placed in a subcutaneous pocket in the back of the neck of the recipient, donor mouse (the same mouse that follicles were collected). After 14 days the grafts were processed and embedded in paraffin blocks, then serially sectioned for histological evaluation. We counted the follicles and classified them according to stage (preantral or antral). Data were presented as mean ± standard error of mean (SEM) and analysed by analysis of variance (ANOVA) and the Kruskal-Wallistest.

**Results:**

The mean percentage of recovered follicles encapsulated and transplanted in each group were 33.30 ± 2.47 (fibrin gel), 31.96 ± 1.90 (fibrin gel+5% PL), 34.02 ± 2.44 (fibrin gel+10% PL), 48.31 ± 2.06 (fibrin gel+15% PL) and 17.60 ± 2.79 (fibrin gel+20% PL). There was a significant increase in the recovery rate of grafted follicles with fibrin gel+15% PL (48.31%; p<0.001). The percentage of preantral follicles showed no significant difference in all groups (p<0.05). The percentage of antral follicles showed a significant decrease in follicles grafted with fibrin gel+20% PL when compared to the other groups (11.77%; p<0.005) but no significant difference was observed in the other groups.

**Conclusion:**

The use of PL in follicle transplantation can improve ovarian follicular survival rate.

## Introduction

Recent improvements in cancer treatments have signiﬁcantly increased survival rates. Unfortunately treatments such as chemotherapy and radiotherapy can cause severe damage to the ovaries and mature ovarian failure. Ovarian cryopreservation prior to chemotherapy/radiotherapy and autotransplantation post-treatment can restore fertility to cancer patients. Options for fertility preservation in patients at risk for premature ovarian failure ( POF ) include embryo cryopreservation, oocyte cryopreservation and ovarian tissue cryopreservation followed by subsequent transplantation (1). 

The major impediment to transplantation is that the graft is completely dependent on the establishment of neovascularization, thus a signiﬁcant ratio of follicles may be lost because of ischemia and hypoxia. Ischemia in the transplant from delayed revascularization is one of the transplantation concerns. In the absence of direct vascular anastomosis, the success and survival of ovarian grafts is dependent upon vascularization. In the mouse, revascularization begins during the ﬁrst 48 hours after transplantation (2). Hypoxia-induced delayed revascularization in the early stages of ovarian transplantation causes the loss of large numbers of immature follicles. On the other hand, numerous growth factors such as platelet-derived growth factors ( PDGF ) (3), vascular endothelial growth factor ( VEGF ), insulin-like growth factor ( IGF ) and basic ﬁbroblast growth factor ( bFGF ) (4,5) involved in neovascularization and follicular growth can be used to improve transplantation. Minimizing the initiation duration of revascularization can improve graft function and follicular survival rate, and reduce premature follicle depletion. 

As an alternative strategy, cryopreservation and transplantation of isolated ovarian follicles has a number of potential advantages over ovarian tissue transplantation. First, ovarian tissue has a more complex structure with different cell types making the choice of a suitable cryopreservation protocol challenging. Cryopreserved-thawed follicles can be suspended in a plasma clot (6,7) or collagen gel (8) permitting auto-transplantation via a minimally invasive approach. Isolated ovarian follicle transplantation can negate the risk of cancer recurrence that is of concern in auto-transplantation of cryopreserved-thawed ovarian tissue in cancer patients in remission (9). 

Extracted activated platelets [platelet lysate ( PL )] are a rich source of angiogenic and growth factors (10,11) which may have an important role in improving transplant survival. Fibrin gel due to its availability, biodegradation and biocompatibility has been used as a delivery vehicle and suitable preservative for transplantation of pancreatic islets (12), muscle-derived stem cells (13) and keratinocytes (14). In this study, as an alternative method, we have autologously transplanted isolated preantral follicles by using ﬁbrin gel supplemented with PL and analyzed the results. 

## Materials and Methods

### Experimental design

In this experimental study, female Naval Medical Research Institute ( NMRI ) mice ( Pasteur Institute, Tehran, Iran ) were housed and bred at the Laboratory Animal Breeding Center of Royan Institute ( Tehran, Iran ). Animals were kept at a temperature of 19-22˚C and 50% humidity un˚ der light-controlled conditions ( 12 hours light: 12 hours dark ) and provided sterile food and water ad libitum. The experiments were approved by the local Ethics Committee at Royan Institute. 

One of the ovaries ( right ovary ) was taken from 14-day-old female NMRI mice after they were anesthetized with ketamine/xylazine ( Sigma, Germany ). A total of 1919 preantral follicles were dissected from the ovaries and divided into ﬁve groups: I. encapsulated into ﬁbrin gel ( group 1; 24.31 follicles per mouse ), II. ﬁbrin gel+5% PL ( group 2; 21.52 follicles per mouse ), III. ﬁbrin gel+10% PL ( group 3; 22.41 follicles per mouse ), IV. ﬁbrin gel+15% PL ( group 4; 22.55 follicles per mouse ) and V. ﬁbrin gel+20% PL ( group 5; 22.17 follicles per mouse ). The follicles were then transplanted back into the same donor mice. Grafted encapsulated follicles were harvested after 14 days and histologically evaluated for survival and development to the next stages. 

### Platelet preparation

To prepare PL, 350-400 ml of human umbilical cord blood ( UCB ) was centrifuged at 300 g for 22 minutes at room temperature. Harvested platelet rich plasma ( PRP ) was centrifuged again at 300 g for 30 minutes at room temperature to obtain the platelet concentrate ( PC ). Harvested PC was stored at -70˚C until use. Then, samples that contained approximately 1×10^9^platelets/ml were selected and frozen at -70˚C to obtain PL that contained platelet-released growth factors. Frozen PC was rapidly thawed at 37˚C and centrifuged at 3000 g for 30 minutes at 4˚C to remove the platelet bodies. 

### Collection of preantral follicles

Mice were anesthetized by ketamine and xylazine. After disinfecting the dorsal skin, a small incision was made and the ovaries removed. Preantral follicles were mechanically collected from ovaries as described previously (15). Briefly, for mechanical isolation, preantral follicles were dissected using a 29 gauge needle attached to 1ml syringe in alpha-minimum essential medium ( α-MEM, Gibco-Invitrogen, Genmany )+10% fetal bovine serum ( FBS, Sigma, Germany ) at 37˚C. Round-shaped preantral follicles with 2-3 layers of granulosa cells and the spherical oocyte centrally located within the follicle were considered to be morphologically normal and used in the experiments. 

### Follicle encapsulation and transplantation

Fibrinogen and thrombin solutions were prepared from Cell Science Research Center of Royan Institute ( Tehran, Iran ). 

For group 1, preantral follicles were suspended in 10 μl of thrombin solution and mixed with 5 μl of reconstituted ﬁbrinogen solution to form the ﬁbrin gel. For group 2, preantral follicles were suspended in a mixture of 9.5 µl of thrombin and 0.75 µl of PL followed by the addition of 4.75 µl of ﬁbrinogen solution ( ﬁbrin gel+5% PL ). For group 3, preantral follicles were suspended in a mixture of 9 µl of thrombin plus 1.5 µl of PL and mixed with 4.5 µl of ﬁbrinogen solution ( ﬁbrin gel+10% PL ). Group 4 preantral follicles were suspended in a mixture of 8.5 µl of thrombin and 2.5 µl of PL, then mixed with 4.25 µl of ﬁbrinogen solution ( ﬁbrin gel+15% PL ). For group 5, preantral follicles were suspended in a mixture of 8 µl of thrombin and 3 µl of PL, after which 4 µl of ﬁbrinogen solution was added ( ﬁbrin gel+20% PL ). In all groups, after 3 to 5 minutes, the ﬁbrin gel/ﬁbrin gel supplemented with PL that contained preantral follicles were placed in a subcutaneous pocket in the posterior area of the mouse’s neck. Each mouse received the follicles that were collected from it. The incision was sutured with polyethylene suture 6 ( Ethicon, USA ) and mice were allowed to recover. After waking up, the mice were transferred to the animal house and kept for 14 days under standard conditions. 

### Histological assessments

After two weeks, the mice were anesthetized and an incision was made in the posterior sections of their necks. Grafted follicles associated with their encompassing tissue were excised and immediately placed in Bouin’s solution. For all groups the ﬁxed follicles were dehydrated and embedded in parafﬁn wax ( Merck, Germany ) serially sectioned in 6 μm sections and stained with hematoxylin–eosin ( H&E ) for histological evaluations. Each ﬁve sections were observed and analyzed by light microscope ( Nikon, Japan ) after which the numbers of morphologically normal follicles were counted on the basis of Gougeon’s study (16). Eosinophilia of the ooplasm, contraction and clumping of the chromatin material, and wrinkling of the nuclear membranes of the oocytes were regarded as signs of atresia (16). The follicles were classiﬁed according to stage as preantral or antral follicles. Preantral follicles were conﬁrmed by the presence of two or more layers of cuboidal granulosa cells without antrum. Antral follicles were identiﬁed as having an antral cavity (17). 

Data were presented as mean ± standard error of mean ( SEM ) and analysed by analysis of variance ( ANOVA ) and the Kruskal-Wallis test. A probability of p<0.05 was considered statistically signiﬁcant. Statistical package for the social sciences ( SPSS ) software ( version 16; SPSS Inc., Chicago, IL, USA ) was used for data analysis. 

### Results

**Fig.1:**
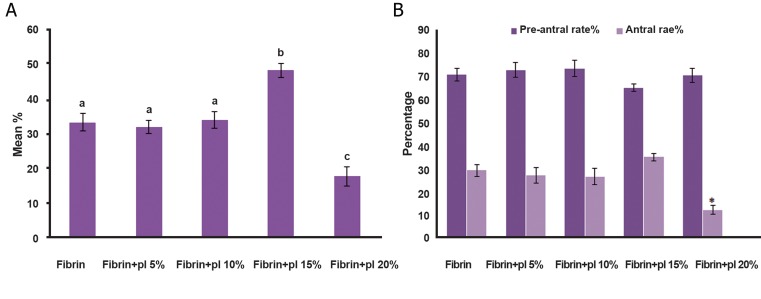
Histological evaluation and comparison of follicular recovery rate. Number of follicles transplanted to the under skin of the posterior neck and number that survived. A. Preantral and antral rate and B. Between grafted isolated preantral follicles with fibrin gel and fibrin gel supplemented with platelet lysate (PL) at various doses, transplanted for 14 days under the skin in the posterior area of the neck. *; Significntly diffrent from other groups in antral rate (p<0.05) and Significant difference between non-identical letter (p<0.01).

We removed ﬁbrin clots that contained isolated preantral follicles from the mice at 14 days after grafting. The follicles were identiﬁed after histological examination of the grafted ﬁbrin clots. Serial histological sections were analyzed to determine follicle number and stage. The total number of follicles encapsulated and transplanted in each group were 389 ( ﬁbrin gel ), 366 ( ﬁbrin gel+5% PL ), 381 ( ﬁbrin gel+10% PL ), 406 ( ﬁbrin gel+15% PL ) and 377 ( ﬁbrin gel+20% PL ). The mean percentages of recovered follicles were 33.30 ± 2.47 ( ﬁbrin gel ). 31.96 ± 1.90 ( ﬁbrin gel+5% PL ), 34.02 ± 2.44 ( ﬁbrin gel+10%PL ), 48.31 ± 2.06 ( ﬁbrin gel+15% PL ) and 17.60 ± 2.79 ( ﬁbrin gel+20% PL ) per grafted ﬁbrin gel for each group (Table 1,Fig.1A). A comparison between the ﬁve groups showed a signiﬁcant increase in the recovery rate of grafted follicles encapsulated with ﬁbrin gel+15% PL ( 48.31%; p<0.001 ) and a signiﬁcant decrease in grafted follicles with ﬁbrin gel+20% PL compared to the other groups ( 17.60%, p<0.001, Fig.1A ). 

The percentage of preantral follicles showed no signiﬁcant differences in all groups ( p<0.05 ). However the percentage of antral follicles signiﬁcantly decreased in the grafted follicles with ﬁbrin gel+20% PL compared to the other groups ( 11.77%, p<0.005 ) but no signiﬁcant difference was observed in the other groups (Fig.1B). 

**Table 1:** Number of transplanted follicles and survived follicles, preantral/antral follicles 14 day post transplantation


Variables	Number of transplanted follicles	Number that survived	Number of preantral follicles	Number of antral follicles

**FG**	389	128	90	38
**FG+5% PL**	366	116	83	33
**FG+10% PL**	381	129	93	36
**FG+15% PL**	406	198	127	71
**FG+20% PL**	377	67	56	11
**N**	1919	638	449	189


FG; Fibrin gel, PL; Platelet lysate and N; Number.

**Fig.2:**
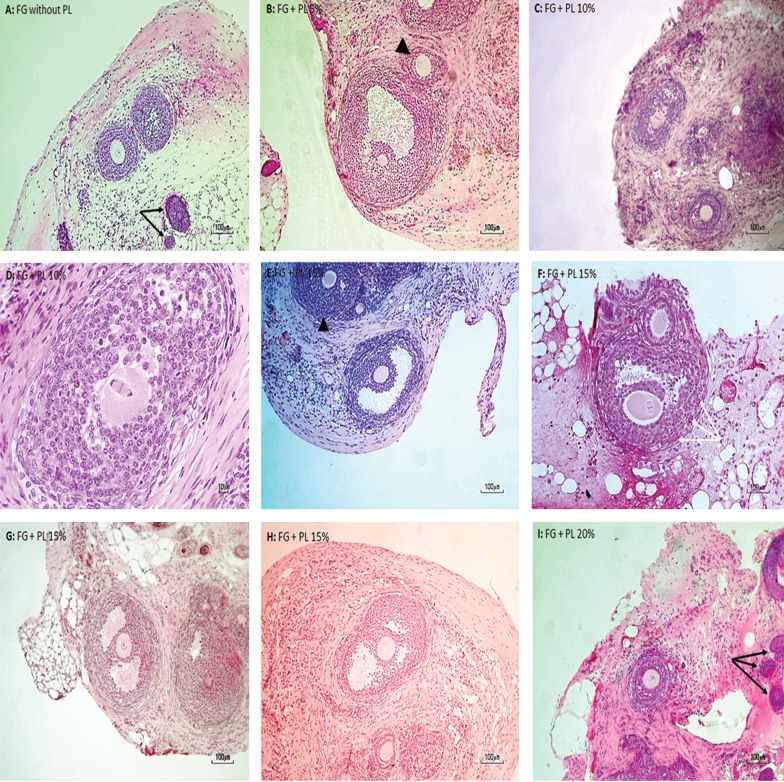
Histologic sections of murine isolated follicle grafts auto-transplanted under the skin in the posterior neck for 14 days. Hematoxylin and eosin ( H&E ) section of murine skin tissue ( around the follicles ), preantral follicles [A, B ( arrow head ), E ( arrowhead ), I] and antral ( B, D, E, H ) follicles in the graft. Original magnification: A-I: ×100; D: ×200. ( Black arrows: hair follicles, white arrows: follicular blood vasculatures ). FG: Fibrin gel and PL: Platelet lysate.

Morphologically, follicles at all stages looked well preserved (Fig.2). Preantral follicles appeared as rounded structuresthat contained intact oocytes with large nuclei, and spherical or slightly ovoid in shape. The oocyte was surrounded by more than two layers of granulosa cells (Fig.2A,B,E,I). Healthy-looking antral follicles also appeared as rounded structures. The fluid-ﬁlled antral cavity of the antral follicle was encapsulated by numerous layers of granulosa cells and some theca cells (Fig.2B,D,E,H). The oocyte with its nucleolus was clearly visible and surrounded by an intact zonapellucida. 

### Discussion

Loss of follicular pool is caused mostly by ischemia and not providing a rapid the blood supply. Isolated follicles due to their lack of stroma may connect faster into newly formed vasculatures. Therefore, in this study isolated follicles have been used for auto-transplantation. For this purpose, the ﬁbrin gel used in this study ﬁxed the follicles at the transplantation site due to its sticky nature and connected the host tissue and graft to each other. This might enhance revascularization via facilitation of cell inﬁltration from the host tissue and paracrine interactions. 

Although the role of angiogenic cytokines such as VEGF, PDGF and FGF in survival, migration and proliferation of endothelial cells is well known, several observations have shown that the extracellular matrix ( ECM ) is equally important (18). To promote migration, cytokine function is entirely dependent on the endothelial cell binding to the ECM which is important during the growth of new blood vessels from existing vessels. Biomaterials are effective in other systems to increase angiogenesis (19). In this study ﬁbrin gel, as the biodegradable scaffold, has been used to maintain the follicles at the graft site. 

PL is a concentration of human platelet growth factors in a small volume of plasma obtained by lysing the platelet bodies through temperatureshock. Therefore, PL contains all the fundamental growth factors secreted by platelets to initiate tissue regeneration and angiogenesis, including PDGFs, bFGF, VEGF, IGF-1 and transforming growth factor-b ( TGF-b ) (10,20). Allare potent angiogenic factors and endothelial cell mitogens. 

**Fig.3:**
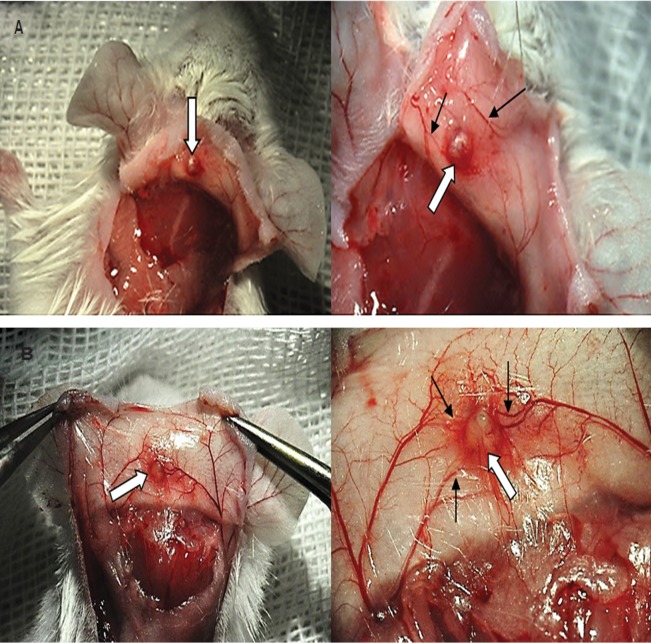
Morphological evaluation of neovascularization in follicle grafts after 14 days. White and black arrows demonstrate grafted follicles and blood vasculature around the grafts, respectively. A. Grafted follicles encapsulated in fibrin gel without platelet lysate (PL) and B. Grafted follicles encapsulated in fibrin gel supplemented with 15% PL.

In this study, we observed that isolated follicles survived and grew in non-capsulated sites such as subcutaneous spaces. The survival rate in group 4 ( fibrin+15% PL ) compared with group 1 ( fibrin gel ) was associated with a significant increase. This increased survival was probably due to the presence of a certain dose ( 15% ) of PL and appeared to be the result of a decrease in ischemia conditions due to the presence of angiogenic and growth factors which led to the provision of a more rapid blood supply to the graft. However, growth and maturation ( antral to the preantral ) were not significantly different between the two groups. Hence, it appeared thatangiogenic and growth factors in PL could only have increased angiogenesis and sprouting new blood vessels (Fig.3). 

This study aimed to evaluate whether transplantation of isolated ovarian follicles encapsulated in fibrin supplemented with PL might have biological properties appropriate for use in fertility preservation. Our results were consistent with a study conducted by Dolmans et al. (7) which reported that loss of grafted follicles ( approximately 50% ) was related to ischemia and delayed revascularization. It seems, although revascularization in transplanted follicles encapsulated in fibrin gel supplemented with PL was facilitated, the lack of stromal cells around the grafted follicles caused no observed significant difference in survival rate to the same another studies (7,21). 

After transplantation the follicles encapsulated in ﬁbrin gel supplemented with PL ( 15% ) had a higher survival rate than follicles encapsulated in PL-free ﬁbrin gel ( 48 vs. 32% ). This indicated that PL which contained angiogenic and growth factors caused more rapid revascularization than group 1 which did not use PL. We showed that different doses of PL added to the ﬁbrin had different effects on follicular survival rate. Survival rates increased signiﬁcantly at the 15% dose compared to the 5, 10 and 20% doses. 

We observed significant differences in the preantral and antral follicle rates after transplantation in each group, which indicated that PL despite the positive role in revascularization and follicular survival did not have a significant impact in follicular growth and maturation. In our experiment the concentrations of growth factors in PL at specified times were not controlled, thus the growth factor concentrations could be reduced over time through release to the environment and their presence could be limited. They might not have been able to influence the final stages of follicular growth. During the first few days after transplantation of preantral follicles we observed a positive effect on survival rate and revascularization, but we did not observe any significant effect in follicular growth and antral follicle rate. 

### Conclusion

Due to the widespread biological effects of this substance whether or not the follicles grafted have developmental potential and also identiﬁcation of possible side effects of this product more research should be done. According to the results the use of PL in ovarian follicle transplantation could improve survival of the follicles. 
